# Early Human Migrations (ca. 13,000 Years Ago) or Postcontact Europeans for the Earliest Spread of* Mycobacterium leprae* and* Mycobacterium lepromatosis* to the Americas

**DOI:** 10.1155/2017/6491606

**Published:** 2017-11-09

**Authors:** Samuel Mark

**Affiliations:** Department of Liberal Studies, Texas A&M University at Galveston, P.O. Box 1675, Galveston, TX 77553-1675, USA

## Abstract

For over a century, it has been widely accepted that leprosy did not exist in the Americas before the arrival of Europeans. This proposition was based on a combination of historical, paleopathological, and representational studies. Further support came from molecular studies in 2005 and 2009 that four* Mycobacterium leprae* single-nucleotide polymorphisms (SNPs) and then 16 SNP subtypes correlated with general geographic regions, suggesting the* M. leprae* subtypes in the Americas were consistent with European strains. Shortly thereafter, a number of studies proposed that leprosy first came to the Americas with human migrations around 12,000 or 13,000 years ago. These studies are based primarily on subsequent molecular data, especially the discovery of a new leprosy species* Mycobacterium lepromatosis* and its close association with diffuse lepromatous leprosy, a severe, aggressive form of lepromatous leprosy, which is most common in Mexico and the Caribbean Islands. A review of these and subsequent molecular data finds no evidence for either leprosy species in the Americas before the arrival of Europeans, and strains of both species of leprosy found in eastern Mexico, Caribbean Islands, and Brazil came from Europe while strains found in western Mexico are consistent with their arrival via direct voyages from the Philippines.

## 1. Introduction

The proposition that leprosy did not exist in the Americas before the arrival of Europeans has been widely accepted for well over a century [1–8]. The Spanish and Portuguese as well as the African slaves they imported have been cited as original sources of leprosy in different regions, including the southern United States, Caribbean Islands, and Central and South America. Northern Europeans introduced a few small pockets of leprosy to North America, like New Brunswick, Canada, and later in the Midwestern United States, while migrant workers from China and India were also later sources of leprosy. These studies (henceforth postcontact theory) have been based on a combination of historical, paleopathological, and representational data. In 2005 Monot et al. discovered that four* Mycobacterium leprae* single-nucleotide polymorphisms (SNPs) correlated with general geographic regions [9], and in 2009 Monot et al. defined 16 SNP subtypes. They then concluded that type 4 strains that are now found in the West Indies and Central and South America spread to these regions via the slave trade from Africa about 500 years ago. In regard to type 3 strains, they state that “it seems unlikely that leprosy was introduced into the Americas by early humans via the Bering straits; rather, it appears more probable that it was brought by immigrants from Europe, as most of the* M. leprae* strains found in North, Central and South America have the 3I genotype found in European leprosy cases. This interpretation is consistent with paleological findings because skeletons with signs of leprosy are limited to the postcolonial period” [10]. Shortly thereafter, a number of papers were published challenging this postcontact theory, and they are primarily based on additional molecular studies, especially the discovery of a new leprosy species,* Mycobacterium lepromatosis*. While these studies present what appears as a viable argument for the spread of both species of leprosy to the Americas around 13,000 years ago, a review of the historic, paleopathological, and molecular data argues against such an early arrival for either species with a modified postcontact theory being consistent with the present evidence.

## 2. Support for an Asian Migration Theory

Matsuoka et al. were the first to raise a challenge to a postcontact theory. When testing for both SNP types and variations of 6 base tandem repeats in the* rpoT* gene (*rpoT* profile), they found that SNP types 3 and 4 of the* M. leprae* species from the Mexican east coast and Yucatan Peninsula had a 3* rpoT* profile consistent with European and African populations, supporting a postcontact theory. In contrast, they found that* M. leprae* SNP types 1, 2, and 3 in western and southwest Mexico had both the 3 and 4* rpoT* profiles. Matsuoka et al. concluded that since the SNP type 2 of the 4* rpoT* profile was dominant in Japan and South Korea, an Asian population migrated to Japan from Korea, while other contemporary Asian populations with this profile migrated across the Bering Strait to western Mexico [11, 12] (henceforth Asian migration theory).

Roa and Morris noted that instead of an Asian migration the* M. leprae* strains from Asia might have spread through the Philippines to Mexico, and they state that “it is commonly said that leprosy was introduced to Mexico from the Philippines during the Spanish Colonial era.” They also noted that these routes and periods of transmission are not mutually exclusive, supporting an initial introduction of leprosy strains c. 12,000 years ago and a reintroduction of new strains during colonial times as suggested by Matsuoka et al. In parallel, Roa and Morris pointed out that in 1908 Jesus Chico stated that the indigenous population in Mexico probably suffered from leprosy before the arrival of Hernan Cortes because leprosy was identified by the Spanish in 1519. They also argued that any confusion with other diseases, like vitiligo or leishmaniasis, was unlikely, and they state that “the first leprosarium was opened in Mexico City by the Spaniard conqueror Hernan Cortes at 1521 to 1524. The leprosarium was built in a place called Tlaxpana” [13].

Further support for an Asian migration theory came in 2008 when Han et al. reported a second leprosy causing species,* M. lepromatosis,* in two Mexican patients suffering from diffuse lepromatous leprosy (DLL) a severe form of lepromatous leprosy (LL). DLL is most common in patients from western Mexico and the Caribbean Islands but rarely reported elsewhere in the world. Since* M lepromatosis* was the only species identified in the earliest molecularly confirmed cases of DLL, Han et al. concluded that DLL was caused by* M. lepromatosis,* while other forms of leprosy were caused by* M. leprae*. Furthermore, they noted that* M. lepromatosis* is similar to* M. leprae* in that it is uncultivable and also acquired the ability to bind to Schwann cells but appears more virulent with possibly a shorter doubling time [14].

In 2012 Han et al. tested 87 cases of molecularly confirmed leprosy in Mexico; 55 were caused by* M. lepromatosis*, 18 by* M. leprae*, and 14 by both. All 13 cases of DLL were caused by* M. lepromatosis*. Additionally, more cases of LL were caused by* M. lepromatosis* than* M. leprae*. Thus, in Mexico,* M. lepromatosis* mainly causes LL and was the only cause of DLL, supporting the previous study. They reiterate that DLL is most common in patients from western Mexico and the Caribbean Islands and noted that it had been reported in India, Iran, Malaysia, Hawaii, France, Tunisia, Brazil, and the United States but none were molecularly confirmed [15]. They concluded that their findings supported an Asian migration theory over 12,000 years ago and “leprosy spread along human migration tracks during the past 100,000 years.” [15].

In 2014, Han et al. confirmed cases consisting of one male from Ontario, Canada, infected with only* M. lepromatosis,* while two ethnic Chinese from Singapore were infected with both species [16]. They also tested 96 samples from other countries: 52 from Brazil, 9 from Myanmar, 31 from Malaysia, and 4 from Uganda.* M. lepromatosis* was found in specimens from Brazil and Myanmar. Of the 52 specimens from Brazil, they detected 36 of* M. leprae* (only LL) and seven with* M. lepromatosis* (only tuberculoid leprosy, TL) and three with both leprosy species. Of the nine Myanmar samples, they detected four of* M. leprae* and two of* M. lepromatosis* (all LL). They then proposed that* M. lepromatosis* was the dominant form in Mexico and* M. leprae* was dominant in all other parts of the world [16].* M. lepromatosis* will therefore develop both TL and LL [16], and in a later study four patients with DLL were positive for only* M. leprae* [17]. Thus,* M. lepromatosis* and* M. leprae* can produce all forms of leprosy but both appear to produce different percentages in different populations.

Based on these data, the authors stated that “in Mexico, the century-long record of DLL and the likely dominance of* M lepromatosis* have led us to the hypothesis that the disease came with the first American settlers from Asia around 13,000 years ago. Finding* M. lepromatosis *in Myanmar in this study and in Singapore earlier supports this Asian origin. Finding it in Brazil accords with further American spread from North to Central America, such as Costa Rica, where DLL has been endemic, and to South America, such as the Amazon region of Brazil. The Canadian man infected with* M lepromatosis *had no significant history of exposure or travel to endemic areas, which raises a likelihood of transmission of this agent in Canada, where aboriginal peoples also live” [16].

Subsequently, Han and Silva constructed a phylogenetic tree of several mycobacteria, based on a study of conserved genes. They proposed that the leprosy ancestor adapted to a parasitic lifestyle up to 20 million years ago in an ancestral ape species. Both* M. leprae* and* M. lepromatosis* diverged about 10 million years ago after infecting different ape groups. One species infected the hominid lineage that eventually evolved into modern humans, while the other species infected another hominid species that eventually infected premodern humans about a million years ago then spread globally during human migrations with* M. lepromatosis* arriving in the Americas around 13,000 years ago [18].

There is supporting evidence that a species of primate was the original host for the most recent common ancestor of both leprosy species.* M. leprae* has infected chimpanzees, but whether they were infected in the wild or after capture is unknown [19–21]. Reports exist of wild chimpanzees with nasal discharge possibly caused by infectious diseases, like* M. leprae*, but they have never been examined [21]. Furthermore, some species of monkeys are possible vectors for* M. leprae* with naturally acquired* M. leprae* being reported in two captive sooty mangabey monkeys, and one was a probable monkey-to-monkey transmission [22]. Over 80% of sooty mangabeys inoculated experimentally with* M. leprae* developed leprosy, while rhesus* (Macaca mulatta)* and African green monkeys* (Cercopithecus aethiops)* also appear to be susceptible [22]. A wild-caught cynomolgus macaque from the Philippines has also been reported with a case of spontaneous* M. leprae*, appearing to have been infected before arriving in the United States [23]. While* M. leprae* has not been found in any wild primate populations, no known efforts have been expended to find it. This susceptibility to* M. leprae* in some monkey and ape populations does lend support to Han and Silva's theory of an ape species, and possibly a monkey species, as an early vector.

Singh et al. compared a near-complete genome sequence of* M. lepromatosis *with that of* M. leprae*, and they calculated an even earlier divergence time from the most recent common ancestor at 13.9 million years ago [17]. They also performed a phylogeographic survey based on 227 leprosy biopsies taken from patients in Venezuela (*n* = 77), Mexico (*n* = 64), Mali (*n* = 48), and Brazil (*n* = 33), while five cases were listed only as “Other.” Of these, 221 contained* M. leprae* and only six* M. lepromatosis*. Since these six cases were all from Mexico, they concluded that* M. lepromatosis* was predominant in Central America and possibly evolved there [17], supporting Han and Silva's early date for its arrival to this region and an Asian migration theory.

Finally, the recent discovery of* M. leprae* and* M. lepromatosis* in Eurasian Red Squirrels* (Sciurus vulgaris)* in England, Ireland, and Scotland can be interpreted as support for an Asian migration theory. These same squirrels were confirmed with* M. leprae* SNP subtype 3I on Brownsea Island, England, and they appear to have been infected with it for centuries [24]. Furthermore,* M. lepromatosis* was molecularly confirmed in Eurasian Red Squirrels in Scotland (henceforth the Scottish strain) [25] and later on Brownsea Island and the Isle of Wright, England [26], and then Ireland [24]. Eurasian Red Squirrels were reintroduced to Ireland between 1820 and 1856 [24]. The Scottish strain and the strain infecting the two Mexican patients from western Mexico diverged from their most recent common ancestor about 27,000 years ago, while the Irish strain diverged from the Scottish strain about 200 years ago, which is consistent with the time of their relocation [24]. Thus, this 27,000-year divergence between the Scottish and Mexican strains can be interpreted as resulting from one human population 27,000 years ago being infected with the most recent common ancestor of the two strains of* M. lepromatosis*. This population then split into two migratory groups with one group moving to Northern Europe and the other migrating to the Americas, suggesting that the migrations of* M. lepromatosis*, at least, began later than the 100,000 years as previously concluded.

## 3. Review of These Data

Chico was an internationally renowned specialist on leprosy, and the statement cited by Roa and Morris, dating to 1908, seems to have been first presented in 1901 [27], while subsequent presentations at conferences or in publications are nearly identical to the original statement [28]. He states the following: “The Spaniards when they first came to Mexico in 1519 found leprosy to be prevalent in Anahuac, that is, the valley of Mexico and the surrounding high plains. Hernan Cortes, moved by the sight of so many lepers, erected for their benefit a hospital which he christened ‘Hospital de San Lazero,' in which they were isolated and cared for. Nobody at that time was able to tell when this awful disease first appeared in the country, but every well-informed native told the same story: that it was very old; beyond man's memory. Nor could any of the natives give the least idea as to its, origin.” He goes on to hypothesize that leprosy must have spread to Mexico from the Hawaiian Islands. His rational was that if leprosy spread to Mexico from the North it would not have completely disappeared from these populations, and as Hawaiians were great seafarers, Hawaii was the most likely source [27, 28]. Leprosy, however, appears to have spread to the Pacific Islands very late and is first recorded in the Hawaiian Islands in 1823 [6]. One reason a postcontact theory is still so widely accepted is that no European explorer or any later colonist ever mentioned or described a case of leprosy or any condition that could be confused with leprosy among Native Americans anywhere on the North American continent.

It should also be noted that Chico failed to cite any primary sources. For example, there is no primary textual evidence that Cortes founded a “Hospital de San Lazero.” Chico goes on to state that he visited this hospital before it closed and the patients were moved to San Pablo [27, 28]. The Hospital de San Lazero that he is describing in this passage was founded by Pedro Lopez in 1572, and Percy Ashburn, a contemporary of Chico's, noted that it was a common belief that Cortes founded such a hospital. So, Ashburn traveled to Mexico in 1896 to make an “inquiry as to whether or not Cortes had founded a leper hospital, and I was assured by eminent specialists in Mexican history, among them Sr. Federico Gomez de Orozco of the Department of History of the National Museum of Mexico, Sr. Licenciado Don Ezequiel Chaves, and Dr. Ignacio Alcocer, that he had not” [29]. This does, however, raise a question. If the Spanish were the earliest vectors of leprosy in Mexico would enough individuals have been infected with leprosy to require a specific hospital for them in only 50 years? It is possible; for example, in only 17 years after the first infected individual arrived on the Island of Nauru in the South Pacific approximately 35 percent of the population was infected with leprosy [6].

In regard to the statement that leprosy was already prevalent in Anahuac upon the arrival of Cortes in 1519, the earliest source I have found is again Chico, and later publications citing this statement also cite only Chico [3, 13, 30]. Among them, Scott states that “Chico, the only writer I know of who affirms that the Spaniards at the Conquest of Mexico in 1519 found cases among the natives, was probably misinformed” [3].

Roa and Morris also stated that confusion with another disease like vitiligo or leishmaniasis was unlikely, but they failed to cite any sources to substantiate this opinion. The confusion they mention may have arisen not because 16th century Europeans had difficulty identifying leprosy from other conditions but, instead, is due to a lack of detail in their publications. Symptoms of conditions were rarely recorded in these early texts and the terminology can be vague. Albert Ashmead noted that the earliest mention of a precontact disease in Mexico is by Bernardino de Sahagún in his* Historia general de las cosas de Nueva España*, an ethnographic study of Mesoamerica, which he began to compile in 1545. In it he states that those “who have the disease of lepra generally lose their eyebrows and suffer great hunger” [1]. These are the only two symptoms. The cure for lepra was to take an herbal bath and drink an herbal mixture. If they were not cured, they were segregated. Juan de Torquemada published his* Monarquía Indiana*, a history and ethnography of New Spain, in 1615. In it he lists three conditions suffered by the indigenous people leprosos, bubosos (syphilis), and sarnosos (scabies) [1]. Most modern scholars assume that lepra and leprosos meant modern leprosy, but among the ancient Greeks and Romans* lepra* denoted a scaling skin condition, like psoriasis, while leprosy was* elephantiasis graecorum* [31]. The Spanish in the 16th century used* mal de san lazaros *for leprosy while lepra could be used to denote a number of conditions,* sarna ơ lepra*, possibly scabies, and* empeine ơ lepra* for syphilis [1], while* gran lepra* meant smallpox and* pequeña lepra* was measles [29]. None of the early Spanish texts use* mal de san lazaros *or describe any condition with symptoms consistent with leprosy, raising the question what does lepra mean in these early texts? Ashmead prefers a translation of syphilis [1] while Myron Echenberg prefers smallpox. The latter states that “smallpox ravished New Spain in 1519 returning every ten to twenty years thereafter and carrying 25 to 50 percent mortality of the inflicted” [32]. An advantage to lepra equating with smallpox is that it fits a larger pattern. According to two Spanish historians Pedro de Cieza de Léon (1550, 1553) and Juan de Betanzos (1551) a condition labeled as lepra spread rapidly throughout the Inca population and was a factor in a war of succession prior to the arrival of the Spanish because it killed both the Inca king and his young son. They both believed this epidemic was smallpox [33]. Ashmead also points out that lepra struck the Native Americans along the Pacific coast [1], but there is no evidence to indicate that they were infected with either leprosy or syphilis, while smallpox and measles ravaged Native American communities north of Mexico. In 1636 smallpox and, shortly thereafter, measles spread to New Mexico with many pueblos losing up to 25 percent of their inhabitants [34]. There is simply no historical evidence that leprosy existed in precontact North or Central America. Whereas smallpox and measles once introduced by the Spanish in the Caribbean Islands would spread so rapidly, they would precede the Spanish as described in the texts.

Even in the Caribbean region and South America the historical evidence is consistent with leprosy arriving very late. Acadians are believed to have brought leprosy with them to Louisiana from Nova Scotia in 1755, and in Florida the Spanish attributed the appearance of leprosy to the importation of African slaves in 1775 [30]. Similarly, on Barbados Island, an English colony, leprosy was first reported in 1755 and was attributed to the importation of African slaves [1]. The earliest report of leprosy in the Caribbean is on Jamaica in 1687 [3].

In South America, leprosy is reported earlier but still appears during the postcontact period. The earliest reported cases were among the Spanish in Bogota, Columbia, in 1573 [3]. To the east in Guyana, Suriname, and French Guiana leprosy arrived in the 17th century and is attributed to the African slave trade [3]. With the discovery of Brazil in 1500, there was no mention of leprosy, and leprosy is not mentioned prior to the 17th century. The prevalence of leprosy was the highest at Rio de Janeiro, Bahia, and Recife, which were also the Brazilian ports of entry [3]. While African slaves are typically noted as the main vector for leprosy by the Portuguese, Scott points out that leprosy was still prevalent in both Portugal and Normandy, France. Moreover, French marines from Normandy were regularly brought to Brazil between 1555 and 1700 [3], allowing for the possibility that both the Portuguese and French were more prominent vectors for the spread of leprosy than recorded. Furthermore, Juliano Moreira, a contemporary of Chico and also a physician of international repute, stated that leprosy did not exist in Brazil before the arrival of the Portuguese. He worked with various indigenous Brazilian peoples including “the Tupis, the Krars (Keras), the Goytacazes, the Guerens, the Gucks, the Parecos, the Guaycurus, the Lengoas, and the Aruwacks.” He found no evidence of leprosy or any disease that could be confused with it in any indigenous population [35]. Thus, without Chico's report there is simply no historical evidence for leprosy during the precontact period anywhere in the Americas, and I have been unable to find any textual evidence for leprosy among Native American populations from the beginning of the postcontact period to the present.

In regard to the paleopathological evidence, thousands of skeletons and mummies of Native Americans from the pre- and postcontact periods have been studied with many exhibiting evidence of a wide variety of conditions, but none from the precontact period have exhibited any evidence of leprosy [4, 6, 7]. From the postcontact period only one cranium has been cited as a possible case of leprosy, dating to 1866 [7]. This cranium exhibited osseous lesions described as* “facies leprosa”* [7] (also known as rhinomaxillary syndrome or Bergen syndrome).* Facies leprosa* consists of three pathological changes to facial bones: endonasal inflammatory changes, atrophy of the anterior nasal spine, and atrophy and recession of the alveolar process of the maxilla confined to the incisor region. However,* facies leprosa* alone does not confirm leprosy. Additional changes are required in the postcranial skeleton [36] because some conditions can mimic* facies leprosa*, like syphilis, tuberculosis, leishmaniasis, and cancer [7], and by 1866 both syphilis and tuberculosis were common conditions in the Americas. Thus, no evidence exists for leprosy among Native American populations in all the Americas during the precontact period or even in the postcontact period. Additionally, if leprosy arrived via the Bering Strait it must have spread through Siberia, but leprosy arrived late. The Russians established themselves among the Yakuts along the Lena River in the 1630s [37], and a Russian document from 1827 states that leprosy only recently appeared among the indigenous population [38]. To the south, missionaries in Mongolia stated in 1903 that leprosy did not yet exist there [39].

While leprosy may have been an ancient disease in China, it appears to have been confined to the South. In 1894 Cantlie traveled to China to treat lepers and study the geographic prevalence of leprosy. He found that it was so rare in northern provinces that people believed they were immune from it. Northern Shadong province was an exception where leprosy existed but was still not prevalent, and while Cantlie mentions it existing in pockets along the Yangtze River [40], it must have been rare because in 1929 the Ministry of Public Health of the Republican government stated that leprosy had just begun to penetrate into the Yangtze River Basin and spread to the northern banks of the Yellow River [41].

The earliest evidence of leprosy in Japan is during the Nara period (AD 710–784), and the earliest known leper homes were established in the Kamakura period (AD 1185–1333) [42]. From Japan it spread north to Korea with a possible description dating to 1251, and the earliest detailed description dates to 1433. In 1445 it was only endemic to Jeju Island between South Korea and Japan [43]. Cantlie noted in 1897 that leprosy in Korea was still at a low prevalence in the south and diminished towards the North [40], which is consistent with it being introduced from Japan. Thus, not only is there a lack of any evidence for either species of leprosy in precontact America, the earliest evidence for leprosy in Siberia dates to 1827.

Additional molecular data argues against an Asian migration theory. Schuenemann et al. calculated a divergence time for the most recent common ancestor for all* M. leprae* strains after comparing five* M. leprae* samples from medieval skeletons with 11 modern samples. The modern samples had longer branch lengths from accumulated substitutions, and by calculating the average distances between strains, a strict clock model was calculated of divergence times with a range of 1975–4562 years ago [44]. Shortly thereafter, Singh et al. calculated a divergence time for the most recent common ancestor for all* M. leprae* strains as 3,607 years ago with a range of 2,204–5,525 years ago (95% highest probability density) [17]. So far, the most recent common ancestor has never been found, and as such, if their calculations are correct, all known strains of* M. leprae* date no earlier than about 5,525 years ago. This date is consistent with the present evidence for an origin for* M. leprae* in either East Africa or India about 4,000 years ago from where it spread throughout the world [9, 10, 31, 44]. If so,* M. leprae* could not have spread to the Americas over 13,000 years ago.

Another argument by Han et al. in support of an Asian migration theory was the close association between DLL and* M. lepromatosis*, which they stated in five points [5]. Point number 5 that “reports of DLL in Singapore and Malaysia are consistent with an assumed Asian origin” [15] really have no bearing in that an Asian origin is consistent with both Asian migration and postcontact theories. As noted above, Roa and Morris pointed out that* M. leprae* strains from Asia might have spread through the Philippines to Mexico, and this sea lane was an important and high-volume route. Sailing around South America was an arduous and dangerous voyage. Thus, from 1565 to 1815 the Spanish built most of their ships that sailed between Manila and Acapulco in Manila [45], and these Manila ships carried goods, including slaves, from China, Japan, and sometimes cargoes from as far west as India [45]. If the* M. leprae* strains found in western Mexico came on Spanish ships from Southeast Asia, then* M. lepromatosis* could also have come on the same ships from the same region. All SNP types with both* rpoT* profiles in western Mexico would be consistent with such a wide ranging trade passing through Manila to Mexico. Furthermore, since the* rpoT* 4 profile is reported only from Korea, Japan, Indonesia, and Mexico, while the* rpoT* 3 profile is reported in all populations [46], it allows for the possibility that the* rpoT* 3 profile is ancestral. If confirmed, it would support a later postcontact theory. It should also be noted that immigration from Asian countries has continued. So, some strains of both species could have arrived considerably later than even the colonial period.

In regard to point 1 that the “lack of description of DLL in Spain excludes a Spanish origin of the disease” [15], Han et al. noted that percentages of* M. leprae* forms varied between populations; 90% TL in India and Africa and 90% LL in Mexico, while TL and LL are equally distributed in Southeast Asia [14]. The same appears to be true for* M. lepromatosis*. As previously noted,* M. lepromatosis* produces both LL and DLL in Mexico but only LL in Myanmar and only TL in Brazil. In regard to the Caribbean Islands, I have not yet found any study that has confirmed that these cases of DLL were caused by* M. lepromatosis*, although it would be surprising if only* M. leprae* was the cause. Regardless, subsequent to Han et al.'s publications, as noted above, an isolated population of red squirrels in Great Britain appears to have been infected with both* M. lepromatosis* and also with* M. leprae* SNP subtype 3I, which is the earliest recorded strain of* M. leprae* in Great Britain with a radiocarbon date between AD 415 and 545 [47]. At present either East Africa or India are proposed as the origin of* M. leprae* [10, 44] from where it eventually spread throughout the Mediterranean region and Europe, eventually to Great Britain. If* M. lepromatosis* developed only TL and LL in European populations, it could have spread unnoticed to England concurrently along the same routes as* M. leprae*. It could also have spread to Portugal and Spain from where both* M. leprae* and* M. lepromatosis* spread to the Caribbean Islands and Brazil.

There are also major obstacles to points 3 and 4, which state that, respectively, “the endemic zone of* M. lepromatosis *matches the Mongoloid migration routes and settlements along the Pacific states, rather than the Gulf of Mexico states that became home to Spanish settlers.” The “endemicity of DLL in the Caribbean and Brazilian DLL cases coincides with further Mongoloid spread in Central and South America.”

The Spanish had settlements in the southern United States, Caribbean Islands, and the East and West coasts of Mexico. Additionally, both species of leprosy coexist on the West coast of Mexico and there is no evidence to suggest that 13,000 years ago the first peoples in Mexico avoided the east coast. Thus, if* M. lepromatosis* spread to Mexico 13,000 years ago, it should be uniformly prevalent throughout the country. Instead, it can be argued that Brazil, the Caribbean Islands, and western Mexico are three regions separated by land and sea that were infected separately with* M. lepromatosis*. Also, an Asian vector for the Caribbean Islands seems improbable because, as previously noted, leprosy is first recorded very late in the Caribbean Islands, and it is primarily attributed to the importation of large numbers of African slaves. So many slaves were imported on sugar producing Caribbean Islands in colonial times that 75–95% of populations were African slaves, while most free people were of African descent [48]. No cited connection exists between the present Caribbean populations and an Asian migration. Both species of leprosy could have spread to the Caribbean Islands from Spain and possibly by African slaves. In Brazil the Portuguese, African slaves, and possibly French soldiers from Normandy were vectors for both species. As previously noted squirrels on Brownsea Island and the Isle of Wright, England, were infected with both species of leprosy, and these islands are only about 145 km from Normandy, France. Finally,* M. lepromatosis* is largely confined to the west coast of Central America, which is consistent with it arriving on Spanish ships from the Philippines.

In regard to point 2, which states the “dominance of* M. lepromatosis *and the endemic nature of DLL indicate that this species and disease are deeply rooted in the country” seems to suggest that an extended period of time is a primary factor, but the authors fail to cite any evidence that this species and disease could not have become so prevalent in a few centuries, instead of 13,000 years. If this species and disease have existed in the Americas for 13,000 years, the prevalence of DLL should be roughly the same in Brazil, the Caribbean Islands, and western Mexico, but as previously noted DLL is most common in the Caribbean Islands and western Mexico. It can also be argued that since Brazil and Mexico have considerably larger indigenous populations than the Caribbean Islands and leprosy arrived so late to these islands, Brazil should have higher rates of DLL. Consequently, the five points presented by Han et al. do not support just an Asian migration, while the evidence is consistent with a postcontact theory.

If it can be shown that the Canadian patient who developed DLL from* M. lepromatosis* was infected in Canada by someone from an indigenous population, it would support an Asian migration theory. He appears to have spent his life in central Canada, and he lacked a history of travel to an endemic region [49]. This patient did, however, winter in Florida and took one Caribbean cruise from there, but he remained on the boat. These trips seemed unrelated to his exposure to* M. lepromatosis* because he presented with DLL symptoms too soon after these trips [49], but the incubation period for* M. leprae* can last from as little as six months to as long as ten years with an average period of three to five years before the appearance of symptoms [50]. Furthermore,* M. lepromatosis* seems to be more virulent than* M. leprae* [14], allowing for the possibility of a shorter incubation period and faster development. The patient was also 72 years old when diagnosed, and he died from a non-small cell carcinoma of the lung shortly thereafter [49]. Thus, his age and malignancy may have been additional factors that compromised his immune system, making him more susceptible to infection by* M. lepromatosis* and accelerating his development of DLL. He may therefore have contracted* M. lepromatosis* during one of his trips to Florida, which is close to regions where DLL is more common, or while on his voyage, which traveled through these same regions for DLL. Considering that the only other cases consistent with DLL caused by* M. lepromatosis* in the North American continent are two siblings, living in Minneapolis and originally from Guerrero, Mexico [51], which is in western Mexico, would suggest that the Canadian patient's chances of contracting* M. lepromatosis* were more likely during his travels than while in Canada where no evidence exists for either species of leprosy in any indigenous population. If so, it would also support the contention that DLL in the Caribbean Islands is caused by* M. lepromatosis* and would also suggest that there is another regional factor besides ethnicity influencing the development of DLL, since* M. lepromatosis* contracted in other regions, like Brazil and Myanmar, develop into TL and LL. This pattern is testable. If the* M. lepromatosis* did spread to the Americas from Europe during the postcontact period, then the* M. lepromatosis* strains found in the Caribbean Islands, Brazil, and the Canadian patient should all be closest to the Scottish strain, and the strains on the West coast of Mexico, which are more likely to have come from Asia during postcontact times, should be closest to the Asian strains in Singapore and Myanmar.

## 4. Conclusion

Based on the data presented above* M. lepromatosis* and* M. leprae* diverged from their most recent common ancestor about 13.9 million years ago when each acquired a new host, possibly different species of monkeys or apes or even a rodent vector.* M. lepromatosis* diverged about 27,000 years ago with the Scottish strain in the Indian Ocean region and the Mexican strain in Southeast Asia. Humans were first infected with* M. leprae* sometime after 5,000 years ago somewhere in the Indian Ocean region, which would explain the continuous divergence of SNP types and subtypes from the most recent common ancestor because* M. leprae* was in a new host. Both species probably remained relatively isolated until the introduction of long-distance trade, especially via seagoing ships. From the Indian Ocean region humans eventually carried both species of leprosy to Europe and then the Caribbean Islands and Brazil while from Southeast Asia through the port of Manila ships carried humans infected with different strains of both leprosy species.* M. leprae* may be the dominant species in most human populations, hiding a greater prevalence of* M. lepromatosis* except in populations where it develops into DLL. This scenario is consistent with present publications on the spread of leprosy throughout the world. However, as noted above, this scenario is testable; the Scottish strain of* M. lepromatosis* should be dominant in Brazil and should have infected the Canadian patient, while the Mexican strain should be dominant in Myanmar and Singapore. As such, any individual with symptoms consistent with leprosy, modern or ancient, should be tested for both species to clarify the possible extent of* M. lepromatosis* as well as the form or forms it produces and the percentage of each within all populations.
